# Supplementary material for: Efficient end-to-end learning for cell segmentation with machine generated weak annotations
Source: Commun Biol. 2023 Mar 2;6:232. doi: 10.1038/s42003-023-04608-5 (PMC9981753; doi:10.1038/s42003-023-04608-5)
Supplement: Supplementary file 3 — Description of Additional Supplementary Data [file 42003_2023_4608_MOESM3_ESM.docx]

**Description of Additional Supplementary Files**

**File name: Fig3b_source_data.csv**

**Description: Tabulated source data for reproducing Fig. 3b.**

**File name: Fig4_source_data.txt**

**Description: Tabulated source data for reproducing Fig. 4.**
